# Waiting times between examinations with intravascularly administered contrast media: a review of contrast media pharmacokinetics and updated ESUR Contrast Media Safety Committee guidelines

**DOI:** 10.1007/s00330-023-10085-5

**Published:** 2023-10-12

**Authors:** Aart J. van der Molen, Ilona A. Dekkers, Remy W. F. Geenen, Marie-France Bellin, Michele Bertolotto, Torkel B. Brismar, Jean-Michel Correas, Gertraud Heinz-Peer, Andreas H. Mahnken, Carlo C. Quattrocchi, Alexander Radbruch, Peter Reimer, Giles Roditi, Laura Romanini, Carmen Sebastià, Fulvio Stacul, Olivier Clement

**Affiliations:** 1 Department of Radiology, Leiden University Medical Center, Leiden, The Netherlands; 2 Department of Radiology, Northwest Clinics, Alkmaar, The Netherlands; 3 Department of Radiology, University Paris Saclay, AP-HP, University Hospital Bicêtre, BioMaps, Le Kremlin-Bicêtre, France; 4 Department of Radiology, University Hospital Trieste, Trieste, Italy; 5 Department of Clinical Science, Intervention and Technology, Unit of Radiology, Karolinska Institutet and Department of Radiology, Karolinska University Hospital in Huddinge, Stockholm, Sweden; 6 AP-HP, Groupe Hospitalier Necker, DMU Imagina, Service de Radiologie, Université de Paris, Paris, France; 7 Department of Radiology, Landesklinikum St Pölten, St Pölten, Austria; 8 Department of Diagnostic and Interventional Radiology, Marburg University Hospital, Marburg, Germany; 9 Centre for Medical Sciences - CISMed, University of Trento, Trento, Italy; 10 Clinic for Diagnostic and Interventional Neuroradiology, University Clinic Bonn, and German Center for Neurodegenerative Diseases, DZNE, Bonn, Germany; 11 Department of Radiology, Institute for Diagnostic and Interventional Radiology, Klinikum Karlsruhe, Karlsruhe, Germany; 12 Department of Radiology, Glasgow Royal Infirmary, Glasgow, UK; 13 Department of Radiology, ASST Cremona, Cremona, Italy; 14 Department of Radiology, Hospital Clinic de Barcelona, Barcelona, Spain; 15 Department of Radiology, Ospedale Maggiore, Trieste, Italy; 16 AP-HP, Hôpital Européen Georges Pompidou, DMU Imagina, Service de Radiologie, Université de Paris, 20 Rue LeBlanc, 75015 Paris, France

**Keywords:** Contrast media, Pharmacokinetics, Renal insufficiency, Hepatic insufficiency, Practice guideline

## Abstract

**Abstract:**

The pharmacokinetics of contrast media (CM) will determine how long safe waiting intervals between successive CT or MRI examinations should be. The Contrast Media Safety Committee has reviewed the data on pharmacokinetics of contrast media to suggest safe waiting intervals between successive contrast-enhanced imaging studies in relation to the renal function of the patient.

**Clinical relevance statement:**

Consider a waiting time between elective contrast-enhanced CT and (coronary) angiography with successive iodine-based contrast media administrations in patients with normal renal function (eGFR > 60 mL/min/1.73 m^2^) of optimally 12 h (near complete clearance of the previously administered iodine-based contrast media) and minimally 4 h (if clinical indication requires rapid follow-up).

**Key Points:**

*• Pharmacokinetics of contrast media will guide safe waiting times between successive administrations.*

*• Safe waiting times increase with increasing renal insufficiency.*

*• Iodine-based contrast media influence MRI signal intensities and gadolinium-based contrast agents influence CT attenuation.*

## Introduction

The pharmacokinetics of contrast media (CM) will determine how long safe waiting intervals between successive CT or MRI examinations should be scheduled. There are few dedicated studies about the optimal time between successive doses of CM in repeated contrast-enhanced studies [1], or when contrast-enhanced CT and contrast-enhanced MRI studies are done in succession.

The purpose of this guideline by the Contrast Media Safety Committee (CMSC) of the European Society of Urogenital Radiology was to review the data on pharmacokinetics of contrast media and to suggest safe waiting intervals between successive contrast-enhanced imaging studies with intravascular iodine-based CM (ICM), gadolinium-based contrast agents (GBCA), or a combination of both.

## Methods

The literature was analyzed using PubMed and Embase databases from January 1975 to May 2022. Multiple repetitive searches with search criteria including synonyms of “pharmacokinetics”, “distribution”, “distribution volume”, “elimination”, “half-life”, “renal insufficiency”, and “hepatic insufficiency” were performed for all contrast media, with languages limited to English and German. Studies were selected by two experienced authors (A.J.v.d.M., I.D.), based on full-text evaluation. Cross-referencing was widely employed. Older studies in-print were acquired via inter-bibliothecarial loans. In total, 90 studies were included in the final review. The concept guideline was discussed and agreed upon at a meeting of the members of the CMSC in June 2022 in Paris (France). A Delphi method was not used in the consensus discussion.

## Results

### Pharmacokinetics and elimination of iodine-based contrast media

Most studies on ICM have employed an open, 2-compartiment model for pharmacokinetic analyses. The first compartment is the plasma in which the molecules are being diluted and the second compartment is the extracellular space of the tissues where there is an effective capillary permeability, i.e. outside the brain. The plasma concentration decays by distribution of the CM from plasma to the extracellular volume (distribution phase, rate constant *α*), and by elimination of the CM from plasma to urine by renal excretion (elimination phase, rate constant *β*).

The elimination phase defines the time when a second intravascular administration of the same or another ICM can be performed safely, with lower risk of accumulation and potential toxicity (such as contrast-associated acute kidney injury). In theory, near-complete elimination to 1.5% of the original concentration is achieved within 6 elimination half-lives (T½ *β*) [2, 3].

#### Results in animal studies

In most animal studies the 2-compartment model describes the pharmacokinetics of ICM well. All ICM behave similarly in early distribution and excretion by glomerular filtration. In animal studies, distribution volumes ranged 180–250 mL/kg, or between 21 and 25% of body weight. This indicates distribution within the extracellular fluid only. Renal excretion is species dependent, and higher for rats, rabbits, and dogs, compared to monkeys and humans due to their higher weight-normalized GFR. Elimination half-life times in rat studies range 20–25 min, in dogs 50–62 min, and in monkeys 71–83 min [4–12].

The excretion in urine within 4 h is 60–85% and within 24 h is 86–95%, depending on the animal species. The urinary excretion is complete within 48 h. Excretion in faeces is species-dependent, less than 1% for dogs and up to 7% for rats [4–12].

#### Results in human studies — normal renal function

Pharmacokinetics in humans can employ the open 2-compartment model. The distribution volume in healthy volunteers and young patients ranges from 165 to 280 mL/kg, indicating a distribution in the extracellular space. Distribution half-lives are short,15–22 min. For current non-ionic ICM, the elimination half-lives are 1.8–2.3 h [2, 13–21], but may already increase to 3.25–4 h in volunteers and patients of older age [22].

Excretion in urine is quick and independent of dose. About 80% of the dose will be eliminated within 4 h, and 93–98% is excreted in 24 h. Faecal excretion is 2–4%. Nonionic ICM are not metabolized, and ICM do not bind to plasma proteins.

The elimination half-lives of high-osmolar ICM that are still in use for fluoroscopy or CT bowel preparation are shorter than for current non-ionic low-osmolar CM used for intravascular administration, in the range of 1.3–1.8 h [7, 23, 24].

#### Results in human studies — renal insufficiency

In patients with renal impairment the half-lives of ICM increase progressively. The literature on pharmacokinetics of currently available ICM in patients with renal insufficiency is scarce and patient categories vary. Using iomeprol 400 mgI/mL, elimination half-lives in patients with mild, moderately, and severely reduced renal function were 3.7 h, 6.9 h, and 15.1 h [16]. For iodixanol 320 mgI/mL, elimination half-lives increased to 23 h in patients with severely reduced renal function [19, 26]. For iopamidol 370 mgI/mL, elimination half-lives in patients with mild and severely reduced renal function increased to 4.2 h and 10.0 h, respectively [25] For iohexol 350 mgI/mL, the elimination half-life in patients with severely reduced renal function was 27.1 h [26]. Based on these data, in moderate renal insufficiency (eGFR 30–60 mL/min/1.73 m^2^), the elimination half-lives increase to a maximum of 6.9 h, while in severe renal insufficiency (eGFR < 30 mL/min/1.73 m^2^) the half-lives vary for several ICM from 10.0 to 27.1 h, depending on the degree of insufficiency. When renal function is impaired, biliary excretion will increase with excretion in faeces up to 8% [19]. Note that the summarized data largely depend on study populations and settings and should be taken as a relative indication.

#### From evidence to recommendation

The physicochemical data of currently used ICM are summarized in Table 1.

**Table 1:** Physicochemical characteristics of iodine-based contrast media

Name	Structure	Ionicity	Application	Concentration	Molecular weight	Osmolality 37 °C	Viscosity 37 °C	1-Butanol/water partition coefficient pH 7.6−37 °C
				(mgI/mL)	(Dalton)	(mOsm/kg)	(mPa s)	*P* (log *P*)
Iohexol	Monomeric	Nonionic	IV	300	821	640	6.1	0.082 (− 1.086)
Iopromide	Monomeric	Nonionic	IV	300	791	607	4.6	0.149 (− 0.827)
Iopamidol	Monomeric	Nonionic	IV	300	777	616	4.7	0.089 (− 1.050)
Iomeprol	Monomeric	Nonionic	IV	300	777	521	4.5	0.105 (− 0.979)
Ioversol	Monomeric	Nonionic	IV	300	807	645	5.5	0.031 (− 1.509)
Iobitridol	Monomeric	Nonionic	IV	300	835	695	6.0	NA
Iodixanol	Dimeric	Nonionic	IV	320	1550	290	11.4	0.043 (− 1.370)
Diatrizoate*	Monomeric	Ionic	Oral	370	614	2150	8.9	0.044 (− 1.356)
Ioxitalamate*	Monomeric	Ionic	Oral	300	644	1710	5.3	NA

*NA*, no data available; *high osmolar contrast medium

Sources: SPCs of individual contrast media; Eloy, Clin Mat 1991; Krause, Invest Radiol 1994; Krause, Invest Radiol 1996; Dawson, Textbook of Contrast Media, 1999; ACR, Contrast Media Manual 2022; Personal communication with Bayer Healthcare, Bracco Imaging, GE Healthcare, Guerbet

For ICM, an open, 2-compartment model is justified and no third compartment for storage can be identified. In patients with normal renal function, the elimination half-lives are 1.8–2.3 h (average 2.0 h). Almost all the administered contrast medium will be cleared in 6 half-lives, 12 h, and already 75% will be cleared in 2 half-lives, 4 h.

In patients with moderate renal impairment (eGFR 30–60 mL/min/1.73 m^2^), renal elimination half-lives increase up to 7 h, so it needs a maximum 42 h for near-complete clearance, and 14 h for 75% clearance. In severe renal impairment (eGFR < 30 mL/min/1.73 m^2^), elimination half-lives vary widely, between 10 and 27 h. In the worst case, it will need a maximum of 7 days for near-complete clearance, and 2.5 days for 75% clearance (Table 2).

**Table 2:** Renal excretion of iodine-based contrast media

Name	Structure	Ionicity	Renal excretion		
			(Elimination T½; hours — near complete elimination in 6 T½)		
			Normal renal function	Moderately reduced RF	Severely reduced RF
			(eGFR > 60 mL/min)	(eGFR 30–60 mL/min)	(eGFR < 30 mL/min)
Iohexol	Monomeric	Nonionic	2.0	NA	27.1
Iopromide	Monomeric	Nonionic	1.8	NA	NA
Iopamidol	Monomeric	Nonionic	2.0	4.2	10.3
Iomeprol	Monomeric	Nonionic	2.3	6.9	15.1
Ioversol	Monomeric	Nonionic	2.1	NA	NA
Iobitridol	Monomeric	Nonionic	NA	NA	NA
Iodixanol	Dimeric	Nonionic	2.2	NA	23.0

*NA*, no data available; *RF*, renal function; *eGFR*, estimated glomerular filtration rate; *T½*, half-life

Sources: see references in text

### Pharmacokinetics and elimination of gadolinium-based contrast agents

Most of the early elimination of extracellular GBCA is via renal excretion; for the hepatobiliary GBCA (gadobenate or gadoxetate), there is additional biliary excretion, which is 4% for gadobenate and 50% for gadoxetate.

The elimination phase defines the time when a second intravascular administration of the same or another GBCA can be performed safely, with lower risk of accumulation and potential toxicity (such as nephrogenic systemic fibrosis or gadolinium deposition). Near-complete elimination to 1.5% of the original concentration is achieved within 6 elimination half-lives (T½ β) [2, 3].

#### Results in animal studies — normal renal and biliary function

All extracellular GBCA behave similarly in early distribution and excretion, except for brain. Elimination half-lives in rat studies range 16–23 min and in rabbit and dog studies 45–60 min for all clinically administered GBCA doses [27–34], with decreases in elimination with increasing age or presence of diabetes of rats [35].

The decrease is first rapid and then progressively slower. Steady-state distribution volumes range 210–230 mL/kg, indicating distribution in the extracellular fluid [27–34]. More than 95% of the contrast is recovered in urine within 24 h after administration. Only small fractions are excreted with bile into the faeces, usually < 4% within 24 h.

For the hepatobiliary GBCA gadobenate and gadoxetate, there is additional biliary excretion that is species-dependent, and high for rats and rabbits. The administration of these CM is associated with a choleretic effect. About 30–35% is eliminated with bile into faeces for gadobenate [29, 33], and 63–68% for gadoxetate [36]. Biliary excretion has a capacity-limiting step with increasing dose, the maximum excretion is about 5 µmol/min/kg.

Brain clearance of macrocyclic GBCA is a slow process, both for cerebrum and cerebellum. Half-lives for elimination were 1.8–2.0 weeks in the first 6 weeks, and 6.3–8.7 weeks thereafter, slightly slower in cerebellum than in cerebrum [37].

#### Results in animal studies — renal and hepatobiliary insufficiency

Only few studies with hepatobiliary GBCA have been performed in rats with combinations of reduced renal and hepatobiliary function. With reduced hepatobiliary elimination, there will be an increased renal elimination and vice versa. Injection of bromosulfophthalein or bile duct ligation can reduce biliary excretion of gadobenate to 1–5%, with concomitant increase in urinary excretion of 66–83% [38]. Renal artery or bile duct ligation reduced elimination half value times of gadoxetate, but significantly more after renal artery ligation. Between 1 and 3% of CM remained in the body in these animals [39, 40].

#### Results in human studies — normal renal and hepatobiliary function

Pharmacokinetic analyses of extracellular GBCA in volunteers showed renal clearances matching the glomerular filtration rate. The reported excretion half-lives range from 1.3 to 1.8 h. Steady-state distribution volumes are in the range of 180–250 mL/kg. Clearance from plasma is rapid with 75–85% of the CM cleared within 4 h, and 94–98% cleared within 24 h [31, 41–47].

For the hepatobiliary CM gadoxetate, the terminal half-life ranged from 1.0 h for young to 1.8 h for older volunteers, with a balanced renal and hepatobiliary excretion. The hepatobiliary excretion is only saturated for high doses, not used in clinical practice [48–50]. Due to its lower hepatobiliary excretion, gadobenate has a profile that is more like the extracellular GBCA. The half-lives were 1.2 h for clinically used doses with distribution volumes of 170–218 mL/kg [51].

#### Results in human studies — renal and hepatobiliary insufficiency

In patients with renal impairment, the half-lives of the extracellular GBCA increase progressively. As in ICM, the summarized data depend on study populations and settings, and should be taken as a relative indication.

In patients with mild renal insufficiency (eGFR 60–90 mL/min/1.73 m^2^), only data for the new GBCA gadopiclenol was available. The half-life already increased to 3.2 h [52]. In moderate renal insufficiency (eGFR 30–60 mL/min/1.73 m^2^), the increase in half-lives was between 3.8 and 6.9 h, depending on the amount of renal impairment, with higher values for lower eGFR. This is equivalent to 2.5–3.5 times that of volunteers with normal renal function. In severe renal insufficiency (eGFR < 30 mL/min/1.73 m^2^), excluding dialysis, half-lives are between 9.5 and 30 h, equivalent to 6–18 times the value of volunteers with normal renal function [52–60].

In the hepatobiliary GBCA, a combination of renal and hepatic impairment has been studied, as bile duct excretion is able to compensate for some renal function deterioration.

Moderate hepatic impairment did not change the plasma half-life, but severe hepatic impairment (Child–Pugh C cirrhosis) led to slight increases to 2.6 h for gadoxetate and to 2.2 h for gadobenate [48, 61]. For gadoxetate, moderate renal impairment could be compensated with a half-life of 2.2 h, while severe renal impairment led to a half-life of 20 h [48]. In gadobenate, moderate renal impairment increased the half-life to 5.6 h and severe impairment to 9.2 h. This is much more like the other extracellular GBCA [57].

#### Results in systematic reviews

In the late 1980s, biodistribution studies suggested that an open, 3-compartment model may better fit the pharmacokinetic data of GBCA than the 2-compartment model [62, 63]. The first compartment is the plasma and the second and third compartments are the extravascular extracellular space of the tissues where there is an effective capillary permeability. The second and third compartments of the model are related to rapidly and slowly equilibrating tissues (storage compartment).

In a large systematic review of pharmacokinetic data, the open 3-compartment model better fitted the data, with 3 phases of GBCA decay from plasma. In addition to the distribution phase (*α*) and rapid (renal) elimination phase (*β*), there is a slow residual excretion phase (*γ*). After IV administration of GBCA, plasma levels of gadolinium fall rapidly, indicating a short distribution phase with an average half-life of 0.2 ± 0.1 h. Then, levels will decrease more slowly as renal elimination prevails, with half-lives of 1.7 ± 0.5 h in plasma [64].

The third phase of decay from the storage compartment could only be demonstrated in urine at a time when concentrations in plasma became undetectable. Rate constant *γ* values are 0.107/h for gadoterate and 0.029/h for gadoxetate, versus 0.007/h for gadodiamide. The half-life for this residual excretion phase is about 5–8 times longer for currently approved linear GBCA (approximately 25 h) compared to a macrocyclic GBCA (6 h), with theoretical risk of dechelation or transmetallation. This residual phase is species-independent, and its rate constant is closely related to the thermodynamic stability of the GBCA molecule.

The relative contribution of this slow elimination is not insignificant, being 21–35% for linear GBCA vs. 10% for macrocyclic GBCA. The exact locations of this third compartment are not completely clear, but Gd retention/deposition can be found in the brain, spleen, liver, kidney, skin, and bones [64].

#### From evidence to recommendation

The physicochemical data of currently available GBCA are summarized in Tables 3 and 4.

**Table 3:** Physicochemical characteristics of gadolinium-based contrast agents

Name	Ligand	Structure	Ionicity	Molecular weight	Osmolality	Viscosity 37 °C	T1 relaxivity in blood, 1.5T^a^	T2 relaxivity in blood, 1.5T^a^
				(Dalton)	(mOsm/kg)	(mPa s)	(l/mmol s)	(l/mmol s)
Gadopentetate	DTPA	Linear	Ionic	939.0	1960	2.9	4.3	4.4
Gadodiamide	DTPA-BMA	Linear	Nonionic	537.6	789	1.4	4.6	6.9
Gadobenate	BOPTA	Linear	Ionic	1058.2	1970	5.4	6.7	8.9
Gadoxetate	EOB-DTPA	Linear	Ionic	682.0	688	1.2	7.3	9.1
Gadoteridol	HP-DO3A	Macrocyclic	Nonionic	558.7	630	1.3	4.4	5.5
Gadobutrol	BT-DO3A	Macrocyclic	Nonionic	604.7	1603	4.9	5.3	5.4
Gadoterate	DOTA	Macrocyclic	Ionic	558.6	1350	2.0	4.2	6.7
Gadopiclenol	NA	Macrocyclic	Nonionic	970.1	843	7.6	12.8	15.1

*EMA*, European Medicines Agency; *Log*, logarithm; *NA*, no data available; *pH*, acidity of a solution; *T½*, half-life

Sources: van der Molen, Eur J Radiol 2008; Port, Biometals 2008; Rohrer, Invest Radiol 2005; Robic, Invest Radiol 2019; Szomolanyi, Invest Radiol 2019; Personal communication with medical departments of Bayer Healthcare, Bracco Imaging, GE Healthcare, Guerbet

**Table 4:** Stability data and constants of gadolinium-based contrast agents

Name	Ligand	Thermodynamic stability	Conditional stability	Kinetic stability	Dissociation constant	Excess ligand	Stability classification
		(pH 14)	(pH 7.4)	(37 °C, pH 1)	Kobs		EMA
		(Log *K*_therm_)	(Log *K*_cond_)	(T½; hours)	(s^−1^)	(mmol/l)	
Gadopentetate	DTPA	22.5	18.4	0.16	0.58	1	Low
Gadodiamide	DTPA-BMA	16.9	14.9	0.01	12.7	25	Low
Gadobenate	BOPTA	22.6	18.4	NA	0.41	0	Intermediate
Gadoxetate	EOB-DTPA	23.5	18.7	NA	0.16		Intermediate
Gadoteridol	HP-DO3A	23.8	17.1	2.0	0.00026	0.5	High
Gadobutrol	BT-DO3A	21.8	14.7	7.9	0.000028	1	High
Gadoterate	DOTA	25.6	19.3	26.4	0.000008	0	High
Gadopiclenol	Piclen	18.7	15.5	120.0	0.000002	NA	NA

*EMA*, European Medicines Agency; *Log*, logarithm; *NA*, no data available; *pH*, acidity of a solution; *T½*, half-life

Sources: van der Molen, Eur J Radiol 2008; Port, Biometals 2008; Rohrer, Invest Radiol 2005; Robic, Invest Radiol 2019; Szomolanyi, Invest Radiol 2019; Personal communication with medical departments of Bayer Healthcare, Bracco Imaging, GE Healthcare, Guerbet

In Europe, for general MRI, only macrocyclic GBCA are allowed. Using the optimized open 3-compartment model, in patients with normal renal function, the renal elimination half-lives are between 1.3 and 1.8 h (average 1.6 h) and the residual excretion time will be in the order of 6 h. Almost all the administered contrast agent will be cleared in 6 half-lives, or 11 h, and already over 75% will be cleared in a little more than 2 half-lives, or 4 h.

In patients with moderate renal impairment (eGFR 30–60 mL/min/1.73 m^2^), the renal elimination half-lives increase to 4–7 h, so it will need up to 42 h for near-complete clearance, and 14 h for 75% clearance. As the residual excretion depends on thermodynamic stability, it will not be significantly prolonged.

For patients with severe renal impairment (eGFR < 30 mL/min/1.73 m^2^), renal elimination half-lives are more prolonged between 10 and 30 h, so it will need up to 60 h (2.5 days) for 75% clearance to 180 h (7.5 days) for near-complete clearance. Thus far, it is unclear if the residual excretion is prolonged in these patients.

For approved linear hepatobiliary GBCA, moderate renal impairment leads to an increase in renal elimination half-lives of 2–5 h, corresponding to 30 h for near-complete and 10 h for 75% clearance. Severe renal impairment leads to an increase in renal elimination half-lives of 10–20 h, corresponding to 60–120 h for near-complete and 20–40 h for 75% clearance. Residual excretion half-lives are in the order of 25 h (Table 5).

**Table 5:** Renal excretion of gadolinium-based contrast agents

Name	Ligand	Structure	Ionicity	Renal excretion		
				(Elimination T½; hours — near complete elimination in 6 T½)		
				Normal RF	Moderately reduced RF	Severely reduced RF
				(eGFR > 60 mL/min)	(eGFR 30–60 mL/min)	(eGFR < 30 mL/min)
Gadopentetate	DTPA	Linear	Ionic	1.6	4.0	30.0
Gadodiamide	DTPA-BMA	Linear	Nonionic	1.3	NA	27.4
Gadobenate	BOPTA	Linear	Ionic	1.2–2.0	5.6	9.2
Gadoxetate	EOB-DTPA	Linear	Ionic	1.0	2.2	20.0
Gadoteridol	HP-DO3A	Macrocyclic	Nonionic	1.6	6.9	9.5
Gadobutrol	BT-DO3A	Macrocyclic	Nonionic	1.8	5.8	17.6
Gadoterate	DOTA	Macrocyclic	Ionic	1.6	5.1	13.9
Gadopiclenol	Piclen	Macrocyclic	Nonionic	1.6–1.9	3.8	11.7

*NA*, no data available; *RF*, renal function; *eGFR*, estimated glomerular filtration rate; *T½*, half-life

Sources: see references in text

### Combined enhanced imaging with an ICM and a GBCA

In oncology, contrast-enhanced MRI examinations with GBCA and contrast-enhanced CT examinations with ICM are often combined, sometimes on the same day. The presence of ICM can influence the (results of the) MRI examination, and the presence of GBCA can influence the (results of the) CT examination. The degree of these effects will determine the optimal order of examinations. The pharmacokinetics of both types of CM will determine how long safe waiting times between examinations should be scheduled.

#### Combining CT and MRI: effects of GBCA on CT studies

Multiple in vitro studies have demonstrated effects of GBCA in CT. At equal mass concentration, GBCA have a higher CT attenuation than ICM due to the higher atomic number of gadolinium (64) compared to iodine (53) [65–72].

Yet, in clinical practice, the molar concentration used for ICM is higher than for GBCA. For instance, iopromide 300 mg I/mL equals 2.94 mmol/mL, compared to GBCA with 0.5–1.0 mmol/mL. Phantom studies focusing on equal attenuation have shown that in CT at 80–140kVp a solution of 0.5 M GBCA is iso-attenuating to a solution of ICM with 91–116 mgI/mL for a chest phantom, and to 104–125 mgI/mL for an abdominal phantom. Due to a different X-ray tube filtration, in DSA at 80–120 kVp, a solution of 0.5 M GBCA is iso-attenuating to 73–92 mgI/mL [73, 74].

Many clinical studies have used GBCA for CT or angiography in renal insufficiency patients or in patients with (severe) hypersensitivity reactions to ICM. The GBCA injection frequently needs high doses of 0.3–0.5 mmol/kg for good vascular enhancement [75], which is relatively short-lived. Such doses may be useful for vascular imaging or interventions, but are not suitable for optimal imaging of the abdominal organs. Good overviews of the results can be found in multiple reviews [76, 77].

Nowadays, such high doses cannot be used anymore. Animal studies have shown that for equal attenuation, GBCA are more nephrotoxic and more costly than low-dose or diluted ICM [74, 78]. In addition to the risk of NSF and gadolinium deposition, these are the major reasons that current ESUR guidelines strongly discourage the use of GBCA for radiographic examinations [79].

Due to the short-lived effect of GBCA enhancement, this vascular enhancement is less cumbersome in clinical CT practice when combining contrast-enhanced CT (or angiography) and MRI examinations on the same day. It must be realized that the kidneys will concentrate GCBA, so that the enhancement of the renal collecting systems, ureters, and bladder may last considerably longer, with risk of misdiagnosis.

#### Combining CT and MRI: effects of ICM on MRI studies

In vitro experiments in MR arthrography may serve as a model for these effects. Mixing of ICM with GBCA will lead to shortening of the T1 (spin–lattice) relaxation time, and a more profound shortening of the T2 (spin–spin) relaxation time. This results in an increase in T1w signal and decrease in T2w signal. The magnitude of the effects is greater for higher GBCA concentrations. The presence of ICM results in higher peak signal intensities at lower GBCA concentrations. In small joint spaces, the overall enhancement was decreased [80–84].

Similar effects are also seen in routine MRI examinations, but to a lesser degree. The shortening effect on T1 and T2 times, with an increase in T1w signal and a decrease in T2w signal, depends on the concentration of the ICM and on the side chains in the molecular structure of the specific ICM that is used (effect is for ioxitalamate > iopamidol > iodixanol, iohexol, or iomeprol) [83, 85–87].

Very recently, in mice, it was shown that adding a high dose of ICM to macrocyclic GBCA leads to a significant increase in r1 relaxation, whereby the combination was excreted more slowly, possibly because of the formation of chemical adducts between the lipophilic three-iodo-benzene rings of the ICM and the tetra-aza-cycle of the macrocyclic GBCA [88]. Increasing concentrations of ICM will also influence diffusion weighted imaging, with increased signal and decreased ADC values [89] and disturb functional imaging with shortening of the T2* times used in BOLD MRI [90].

#### From evidence to recommendation

The effects of ICM are longer-lived and more disturbing on subsequent contrast-enhanced MRI than the effects of GBCA in contrast-enhanced CT. Therefore, it is better to schedule MRI with GBCA before CT with ICM when combining studies. Only for renal imaging CT (including CT urography) is best performed before MRI. The minimum time delay between exams depends on renal function.

## Recommendations of the Contrast Media Safety committee

The new recommendations shown below are based on formal literature review, use more differentiated patient groups, and include recommendations on combinations of imaging studies within a short time interval. The previous recommendations in the electronic ESUR guidelines v10 [91] were based on more limited expert opinion, used fewer patient groups, and focused on 75% excretion or 2 half-lives. For patients with normal renal function, the current minimal waiting times for ICM and GBCA, and for patients with severely reduced renal function, the current minimal recommendations for ICM and the optimal recommendations for GBCA for patients are similar to the previous recommendations.

### Safe time intervals in enhanced imaging with iodine-based contrast media

The CMSC recommends the following waiting times between successive administrations of iodine-based contrast media in contrast-enhanced CT or (coronary) angiography to avoid accumulation of iodine-based contrast media with potential safety issues: 
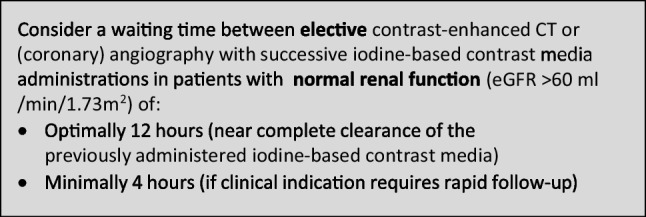

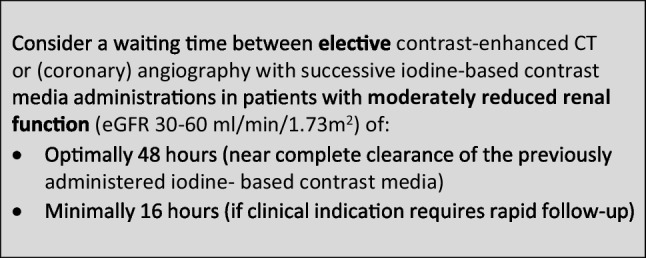

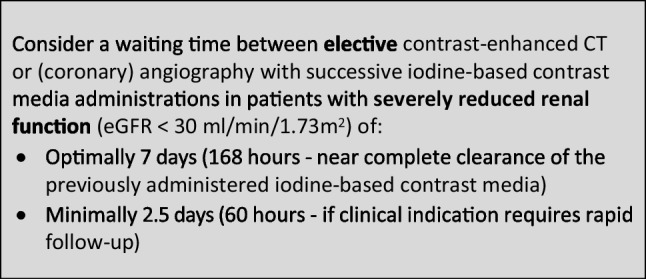

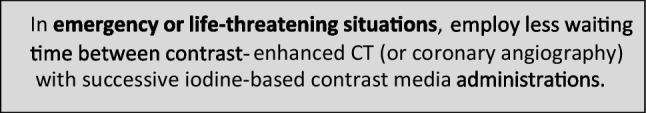


### Safe time intervals in enhanced imaging with gadolinium-based contrast agents

The CMSC recommends the following waiting times between contrast-enhanced MRI with successive administrations of gadolinium-based contrast agents, to avoid accumulation of gadolinium-based contrast agents with potential safety issues: 
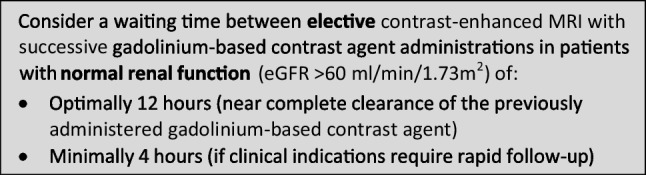

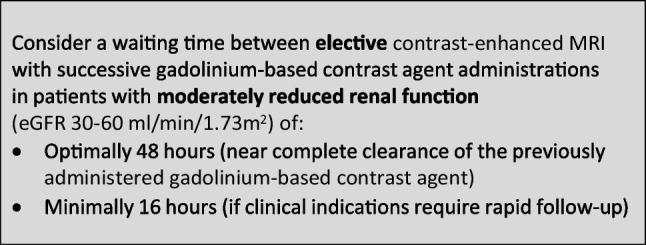

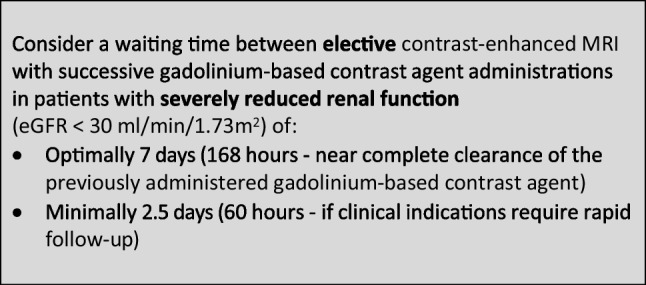

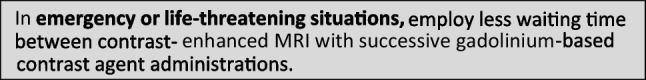


### Safe time intervals in combined enhanced imaging with an iodine-based contrast medium and a gadolinium-based contrast agent

The CMSC recommends the following waiting times between contrast-enhanced MRI and contrast-enhanced CT or (coronary) angiography or vice versa, to avoid interference of the contrast medium used in the first contrast-enhanced examination on the other contrast-enhanced examination, and to avoid accumulation of contrast media with potential safety issues. 
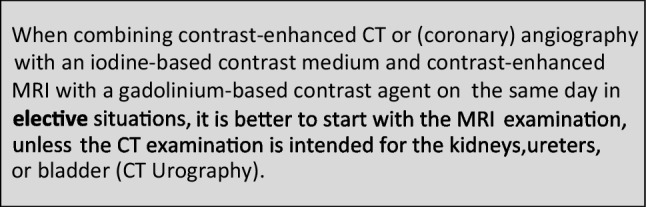


In patients with normal renal function, the interference of the contrast medium used in the first contrast-enhanced examination on the second contrast-enhanced examination witll predominantly determine the suggested waiting times. As the effect is short lived, waiting times can be shorter than for avoidance of accumulation. 
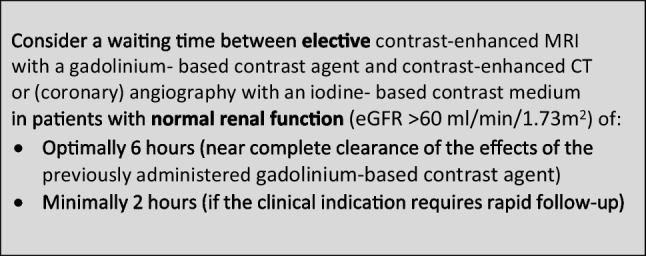


In patients with reduced renal function, the avoidance of accumulation of contrast media with potential safety issues will predominantly determine the suggested waiting times (as in Sects. 1 and 2 above). 
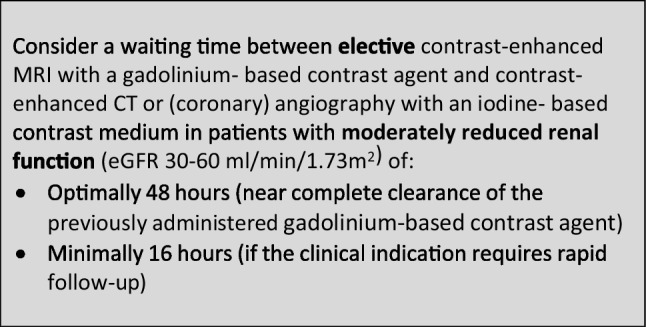

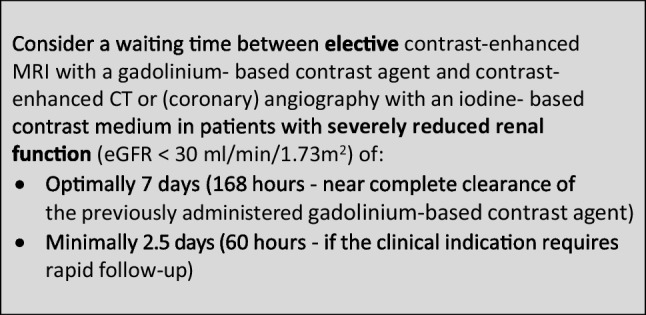


## Abbreviations

CM: Contrast medium/media
CT: Computed tomography
GBCA: Gadolinium-based contrast agent/agents
GFR: Glomerular filtration rate
ICM: Iodine-based contrast medium/media
MRI: Magnetic resonance imaging
T½: Half-life

## Declarations

### Guarantor

The scientific guarantor of this publication is Dr Aart van der Molen.

### Statistics and biometry

Basic statistics were necessary.

### Methodology

• Retrospective, multicentre study
